# Improved recognition of ineffective chest compressions after a brief Crew Resource Management (CRM) training: a prospective, randomised simulation study

**DOI:** 10.1186/s12873-017-0117-6

**Published:** 2017-03-03

**Authors:** Leopold Haffner, Moritz Mahling, Alexander Muench, Christoph Castan, Paul Schubert, Aline Naumann, Silke Reddersen, Anne Herrmann-Werner, Jörg Reutershan, Reimer Riessen, Nora Celebi

**Affiliations:** 1 DocLab, Faculty of Medicine, University of Tübingen, Elfriede-Aulhorn-Straße 10, 72076 Tübingen, Germany; 2 Department of Internal Medicine, Division of Endocrinology, Diabetology, Nephrology, Vascular Disease and Clinical Chemistry, University of Tübingen, Otfried-Müller-Straße 10, 72076 Tübingen, Germany; 3 Department of Anesthesiology and Intensive Care Medicine, University of Tübingen, Hoppe-Seyler-Straße 3, 72076 Tübingen, Germany; 4 Department of Anesthesiology and Intensive Care Medicine, Passau Hospital, Innstraße 76, 94032 Passau, Germany; 5 Institute for Clinical Epidemiology and Applied Biometry, University of Tübingen, Silcherstraße 5, 72076 Tübingen, Germany; 6 Department of Internal Medicine VI, Psychosomatic Medicine, University of Tübingen, Osianderstraße 5, 72076 Tübingen, Germany; 7 Department of Anesthesiology and Intensive Care Medicine, Bayreuth Hospital, Preuschwitzer Straße 101, 95445 Bayreuth, Germany; 8 Department of Internal Medicine, Medical Intensive Care Unit, University of Tübingen, Otfried-Müller-Straße 10, 72076 Tübingen, Germany; 9 PHV-Dialysezentrum Waiblingen, Beinsteiner Str. 8/3, 71334 Waiblingen, Germany

**Keywords:** Crew resource management, Healthcare, Cardiopulmonary resuscitation, Communication, Simulation training, Hospital rapid response team, Emergency medicine

## Abstract

**Background:**

Chest compressions are a core element of cardio-pulmonary resuscitation. Despite periodic training, real-life chest compressions have been reported to be overly shallow and/or fast, very likely affecting patient outcomes. We investigated the effect of a brief Crew Resource Management (CRM) training program on the correction rate of improperly executed chest compressions in a simulated cardiac arrest scenario.

**Methods:**

Final-year medical students (*n* = 57) were randomised to receive a 10-min computer-based CRM or a control training on ethics. Acting as team leaders, subjects performed resuscitation in a simulated cardiac arrest scenario before and after the training. Team members performed standardised overly shallow and fast chest compressions. We analysed how often the team leader recognised and corrected improper chest compressions, as well as communication and resuscitation quality.

**Results:**

After the CRM training, team leaders corrected improper chest compressions (35.5%) significantly more often compared with those undergoing control training (7.7%, *p* = 0.03*). Consequently, four students have to be trained (number needed to treat = 3.6) for one improved chest compression scenario. Communication quality assessed by the Leader Behavior Description Questionnaire significantly increased in the intervention group by a mean of 4.5 compared with 2.0 (*p* = 0.01*) in the control group.

**Conclusion:**

A computer-based, 10-min CRM training improved the recognition of ineffective of chest compressions. Furthermore, communication quality increased. As guideline-adherent chest compressions have been linked to improved patient outcomes, our CRM training might represent a brief and affordable approach to increase chest compression quality and potentially improve patient outcomes.

**Electronic supplementary material:**

The online version of this article (doi:10.1186/s12873-017-0117-6) contains supplementary material, which is available to authorized users.

## Background

Cardiac arrest remains the foremost cause of death in Europe [1]. Cardiopulmonary resuscitation (CPR) and defibrillation represent the core elements of cardiac arrest treatment [2, 3]. The European Resuscitation Council (ERC) guidelines recommend performing chest compressions with a depth of 50 to 60 mm at a rate of 100 to 120 compressions per minute. Studies investigating CPR quality in real-life situations have shown that the chest compression quality (i.e. correct depth and frequency) often is low, even if performed by health-care professionals who have received high levels of cardiopulmonary resuscitation training [4–6]. A low chest compression quality probably negatively affects patient outcome, as shown by experimental and clinical studies [2].

Beyond the regular training recommended by the ERC guidelines [7], there are no reports on how real-life chest compression quality can be improved by Crew Resource Management (CRM) trainings. As resuscitations are usually performed by teams in which the team members perform different tasks, this offers the opportunity for a team leader to recognise and correct incorrectly executed tasks. CRM training programs have already been shown to improve resuscitation quality in terms of a shorter time to defibrillation and a significantly reduced hands-off time [8, 9].

However, at this time, it remains unclear whether the accuracy of chest compressions can be increased by team interventions. We therefore developed an intervention to increase the rate of correctly performed chest compressions and evaluated the effect of this intervention in a standardised realistic cardiac arrest simulation.

## Methods

### Study design, setting and ethics

We carried out a prospective, randomised blinded simulation study. The study took place at the DocLab of the Medical Faculty of the University of Tübingen, Germany, between May and June 2014 as part of a curricular resuscitation course. Students participated voluntarily and were asked to provide written informed consent. Students who did not want to participate in the scientific investigation were offered a comparable training session without data acquisition. We conducted the study in accordance with the Declaration of Helsinki [10], with approval of the ethical committee of the University of Tübingen.

### Participants and randomisation

**Fig. 1:**
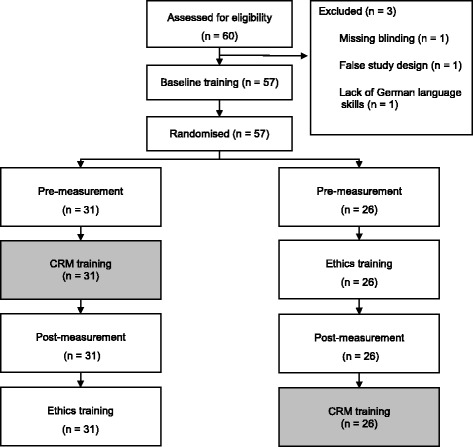
Study design (CONSORT) [23] flow diagram. Sixty participants were recruited. Three participants were excluded from data analysis for the following reasons: lack of blinding (*n* = 1), incomplete scenario (*n* = 1), and lack of comprehension (*n* = 1). The participants drew an envelope for random assignment to each group. This resulted in the randomisation of 31 participants to the intervention group and 26 participants to the control group

All participants were sixth year (final-year) medical students at the beginning of their final year rotations and had successfully finished a practical emergency medicine course as well as an anaesthesiology internship during their third and fourth years. Sixty students were asked to participate in the study (Fig. 1). Three participants were excluded from the analysis because of lack of blinding (*n* = 1), incomplete scenario (*n* = 1) and language barrier (*n* = 1). No student declined participation. The remaining 57 participants were randomised to each group by drawing a sealed envelope (Table 1). Thirty-one participants were randomised into the CRM training group and 26 participants into the control group, which involved an ethics training. An average of six participants were trained each day.

**Table 1:** Baseline characteristics and demographics of participants

	Total	Ethics	CRM
Students (*n*)	57	26	31
Age (median, IQR)	26, 25–29	27, 25–30	26, 25–29
Female (*n* (%))	40 (69.0%)	18 (69.2%)	21 (67.7%)
Previous training in healthcare (*n* (%))	19 (33.3%)	9 (34.6%)	10 (32.3%)
Months since last resuscitation training (mean ± SD)	20.0 ± 8.7	20.0 ± 8.6	20.0 ± 7.6
Regularly resuscitation trainings (*n* (%))	7 (12.2%)	4 (15.4%)	3 (9.3%)
once a year	4 (6.9%)	2 (7.7%)	2 (6.2%)
twice a year	1 (1.7%)	1 (3.8%)	0 (0%)
four times a year	2 (3.4%)	1 (3.8%)	1 (3.1%)
Previous CRM-Training (*n* (%))	9 (15.5%)	3 (11.5%)	6 (18.8%)
Previous Ethics-Training (*n* (%))	8 (13.8%)	2 (7.7%)	6 (18.8%)

### Baseline training

Before the first measurement (pre-measurement), every participant received a training based on basic and advanced life support (ALS). The basic life support training comprised theoretical and practical parts. The training was conducted with either one or two participants. The training focused on the correct execution of the chest compression and ventilation. The participants had to perform these skills on a patient simulator (Resusci Anne Simulator, Laerdal Medical GmbH, Puchheim, Germany) until they were adherent to the guidelines. The advanced procedure training consisted of a theoretical training concerning the application of drugs, defibrillation and advanced airway management.

### Resuscitation scenarios

After the baseline training, every student performed an initial resuscitation (pre-measurement). The participant was presented with two tutors helping him during the resuscitation. The tutors assumed the role of team members while the participant was instructed to take the lead of the team. The scenario was an unresponsive person in a simulated clinical setting. The group was equipped with a medical emergency kit and a defibrillator. The resuscitation scenario lasted approximately 10 min for each student. All students were expected to initiate cardiopulmonary resuscitation and to defibrillate the patient.

### Team members with standardised error

**Fig. 2:**
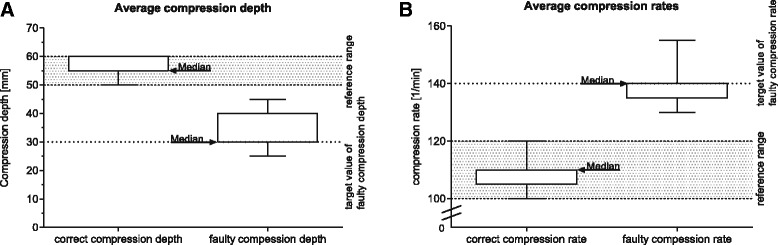
Assessment of the standardisation for compression depth by the tutor. **a**. Correct compression depth: Median (Q25–Q75): 55 (55–60) mm, *n* = 51; incorrect compression depth: 30 (30–40) mm, *n* = 51; and compression rate (**b**). Correct frequency: 110 (105–110) bpm, *n* = 51 and incorrect frequency: 140 (135–140) bpm; *n* = 51 (Box-plot). Before every resuscitation scenario, the tutors performed the correct and the incorrect execution of the chest compressions separately. The hatched area highlights the reference range of the European Resuscitation Council Guidelines 2015 (50–60 mm and 100–120 bpm)

To standardise the study settings, tutors were instructed to perform overly shallow and fast chest compressions. When the tutors were instructed to start chest compressions by the participant (team leader), they performed chest compressions with a median compression depth of 30 mm and a median compression rate of 140 beats per minute (bpm). The standardisation was maintained by measuring the incorrect and correct chest compressions before every scenario, while the participant was not at the scene (Fig. 2).

### CRM and control training

The succeeding CRM training started off by briefly providing participants with information about the importance of CRM in medicine. To develop the four learning objectives, we selected four out of the 15 key points of CRM as formulated by Rall [11]. These objectives were to anticipate and plan, keep everybody informed, communicate effectively and crosscheck. The ethics training explained the four fundamental principles of Medical Ethics from Beauchamp and Childress [12]. The total content of both trainings was chosen to be of a comparable length. Every participant received the missing training after the conduction of the study. Both interventions are available as Additional file 1.

### Outcomes

The primary outcome was defined as the amount of mistakes corrected. The resuscitations were recorded by two cameras, and videos were analysed by two independent video evaluators.

As the secondary outcome, we surveyed the Leader Behavior Description Questionnaire (LBDQ). The LBDQ, used in this study, has been modified by Cooper and Wakelam for the use in resuscitation scenarios [13]. Further secondary outcomes were the compression depth and the compression rate recorded by the patient simulator system. The data of the compression depth and compression frequency were extracted from the log-files and were analysed using an in-house algorithm.

### Statistical analysis

Variables following a normal distribution are presented as mean and standard deviation (±SD). Variables not normally distributed are expressed as median and 25–75% quartiles (Q25–Q75). To analyse the difference in the LBDQ totals, we used the unpaired two-sample *t*-test assuming equal variances. To compare the correction of erroneous chest compressions we used Fisher’s exact test. We assumed a global significance level of 0.05 and corrected for multiple testing using the Bonferroni–Holm approach. Statistically significant results are indicated by an asterisk (*). JMP software (JMP 11.1.1 64-bit version, SAS Institute Inc., Cary, NC, USA) was used to perform statistical calculations, and graphics were drawn using Prism (Version 6.01, GraphPad Software Inc., La Jolla, CA, USA).

## Results

### Demographic data

The demographics and previous experiences of the students are shown in Table 1. All of the participants were final-year medical students. No relevant differences were detected when comparing the CRM and ethics groups.

### Standardisation of incorrect compressions by the tutors

To ensure the standardisation of the scenarios, the tutors (*n* = 7) performed a series of correct and incorrect chest compressions prior to each scenario (Fig. 2). Due to technical problems, six datasets of the standardisation were excluded from analysis. For the chest compression depth, we found a high accordance with the instructions when tutors were asked to perform correct chest compressions (Fig. 2a). Accordingly, when tutors were asked to vary frequency between correct and incorrect chest compression, the adherence of tutors to the instructions was equally high (Fig. 2b).

### Difference of the LBDQ totals

**Fig. 3:**
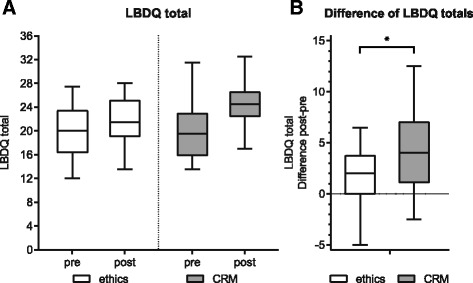
**a** The total Leader Behavior Description Questionnaire (LBDQ) score for the pre- and post-measurement (boxplot). **b** While the mean difference (median pre/post) in the ethics group is 2.0 (20.0/21.5), it is 4.5 (19.5/24.5) in the CRM group

To investigate the effect of the CRM and ethics intervention on communication skills, the LBDQ score was assessed before and after the intervention (Fig. 3a). To evaluate the LBDQ improvement, we calculated the individual difference between the post- and the pre-measurement LBDQ scores (Fig. 3b). The CRM intervention led to a significantly higher increase in the LBDQ total score (4.5 ± 3.4 points) compared with the ethics intervention (2.0 ± 3.9 points, *p* = 0.01*, two-sample *t*-test, Bonferroni–Holm: *p* was significant for *p* < 0.025).

### Correction of erroneous chest compressions

**Fig. 4:**
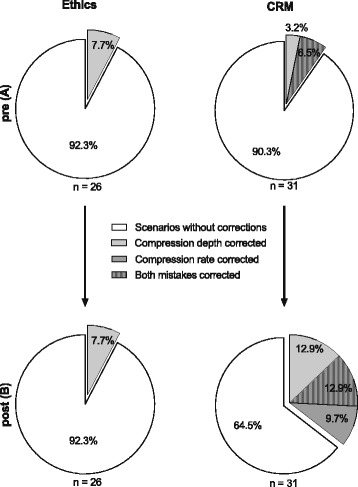
Pie diagram of each group and the pre- (**a**) and post-measurement stages (**b**) for ethics (left) and CRM groups (right). The shaded parts represent the scenarios in which an error was corrected. The white area represents scenarios where neither the compression rate nor the compression depth was corrected. **a** Ethics: compression rate corrected 7.7% (n _of corrections (oc)_ = 2), total: 7.7% (n_oc_ = 2), *n* = 26; CRM: compression depth corrected in 3.2% (n_oc_ = 1), both mistakes corrected in 6.5% (n_oc_ = 2), total: 9.7% (n_oc_ = 3), *n* = 31. **b** Ethics: compression rate corrected in 7.7% (n_oc_ = 2), total: 7.7% (n_oc_ = 2), *n* = 26; CRM: compression depth corrected in 12.9% (n_oc_ = 4), compression rate corrected in 9.7% (n_oc_ = 3), both mistakes corrected in 12.9% (n_oc_ = 4), total 35.5% (n_oc_ = 11), *n* = 31

We investigated the rate of scenarios where the team leader recognised and corrected the improper chest compressions (Fig. 4). Before undergoing the intervention, the rate of scenarios with corrections of both compression rate and frequency was comparable for both groups (Fig. 4a ethics: 7.7%, CRM: 9.7%). After the CRM training, the rate of errors corrected was significantly higher in the CRM group compared with the ethics group (Fig. 4b ethics: 7.7%, CRM: 35.5%; Additional file 2: Table S1 *p* = 0.03*, Fisher’s exact test, Bonferroni–Holm: p was significant for *p* < 0.05). The odds ratio for the correction was 6.6.

During the pre-measurement stage, the ethics training group detected the wrong compression rate only. In the CRM training group, both the wrong compression rate and depth were detected (Fig. 4a). During the post-measurement stage, the ethics training group still recognised the wrong compression rate. In the CRM training group every mistake combination was recognised (Fig. 4b).

### Chest compression

**Fig. 5:**
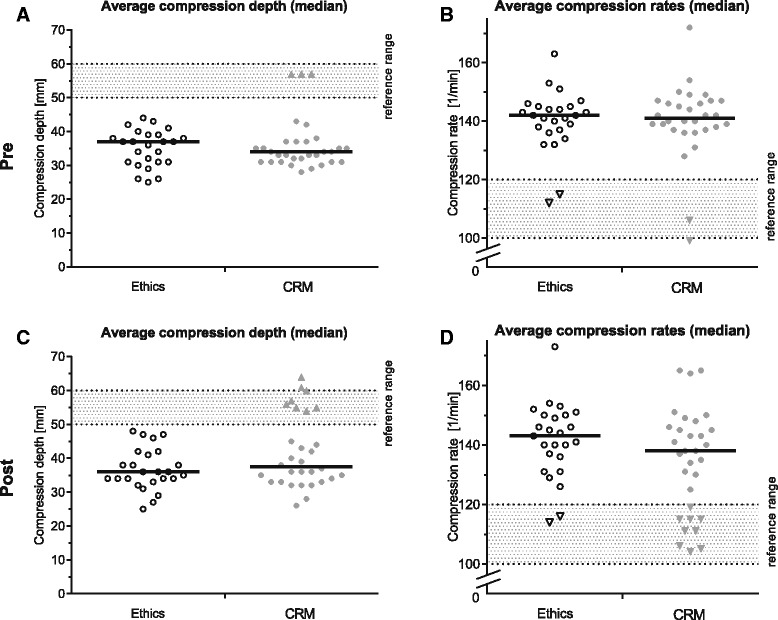
Average chest compression quality during the resuscitation (scatter plot). **a**, **c** Mean compression depth of the resuscitations. The points indicate one resuscitation each, where no correction of the compression depth was applied. A triangle mark shows the average compression depth of the resuscitation after the participant corrected the tutor. **b**, **d** Average compression rates. The triangle indicate that a correction of the compression rate has been applied and the average was calculated based on this correction. **a** Ethics: Median (Q25–Q75) 37.0 (31.0–39.0) mm; CRM: 34.0 (31.0–37.00 mm) **b** Ethics: 142.0 (137.0–145.0) bpm; CRM: 141.0 (137.25–146.75) bpm **c** Ethics: 36.0 (33.5–41.5) mm; CRM: 37.5 (33.0–54.25) mm **d** Ethics 143.0 (133.5–150.0) bpm; CRM: 137.5 (115.0–146.5) bpm

We graphically analysed if the standardised chest compressions (delivered by the tutor) were within the ERC 2015 reference range (Fig. 5, Additional file 3: Figure S1). If the tutors were corrected, we only used the data acquired after the correction to improve readability (indicated by a triangle in the graphic). If no correction was made, we used the data from the beginning to the end of the assessment.

## Discussion

In this study, we prospectively investigated the effect of a short CRM-based training program on the correction rate and resuscitation quality in a cardiac arrest setting involving standardised overly shallow/fast chest compressions. We provide preliminary evidence that even a brief CRM training can result in a significant increase in scenarios in which incorrect chest compressions were corrected.

Originally, principles of CRM have been transferred from aeronautics and aviation into medicine to reduce patient-relevant mistakes caused by human errors [11, 14]. The application of CRM in the context of resuscitation training has already been investigated in several studies. In 1999, Cooper et al. observed 20 resuscitation events in a clinical setting [13]. They showed that good communication and leader behaviour are linked to a better team dynamic and task performance. They also showed that undergoing an ALS training solely could not enhance the leadership performance [13]. In a randomised controlled trial, Fernandez Castelao et al. showed that a CRM training significantly reduced the no-flow time during ALS resuscitation performed by final-year medical students [9]. It is thus not surprising that the importance of CRM trainings has been recognised and recommended in the 2015 ERC guidelines [7]. In the Additional file 4: Table S2, we have summarised the main effects of CRM training on resuscitation-related outcomes in previous studies.

Despite the described positive effects of a CRM training on resuscitation scenarios, it is unknown if these trainings can also improve the recognition of incorrect chest compressions provided by other team members. As most studies investigated ad hoc formed teams (teams that form in the moment they have to perform), this did not enable the investigation of standardised errors performed by team members. Moreover, ad hoc teams face many difficulties; Hunziker showed that these ad hoc teams delayed defibrillation and had a shorter hands-on time [8, 15] (Additional file 5: Figure S2). The hands-on time in Hunziker et al. studies was defined as the duration of time in the first 180 s of resuscitation in which chest compressions are performed. To count as an interruption, a pause had to be longer than 10 s. For each defibrillation, 10 s were added to the hands-on time [15]. We decided that CRM training should be short and computer-based, because personal resources for training purposes are often limited. Moreover, it has been shown that computer-based learning is a good alternative to teacher-focused learning in terms of cost and time efficiency [16, 17].

Because chest compressions have been observed to be overly shallow and/or fast in real-life situations [4], we asked the tutors to perform incorrect chest compressions in a standardised manner. In our study, the incorrect chest compressions were not corrected in about 90% of the scenarios before the intervention. This was similar in both study groups, although more participants in the CRM group had received a CRM training before our study (6 vs. 3 participants). Hunziker et al. stated similar findings among the physicians that they evaluated and concluded that their results were caused by a lack of adherence to the guidelines [15]. Thus, it seems that resuscitation training does not prepare the subjects to recognise or correct wrong chest compressions performed by others.

After the pre-measurement stage, our participants randomly underwent either CRM or ethics training. Consequently, the communication quality, measured using the LBDQ, increased significantly in the CRM group compared with the ethics group. This shows that even a short, computer-based intervention yields a significant effect on communication skills. Hunziker reported similar findings for a 10-min intervention [18]. The communication quality also slightly increased in the ethics groups, most likely representing an effect of training during the pre-measurement stage.

In accordance with an increased LBDQ score, participants in the intervention group were significantly more likely to correct improper chest compressions (odds ratio 6.6). Scenarios without correction were only found in 65% of the cases, compared with 91% at the pre-measurement stage. While this represents a strong effect compared with baseline, it is disappointing that still 65% of the participants neither recognised nor corrected the overly fast or shallow compressions. This might be related to the fact that resuscitation scenarios, even in simulation, pose a significant stress factor to participants that do not have years of experience in emergency medicine [19]. Indeed, simulator studies have further shown that the error rate increases in stress situations [20–22].

In this study, we investigated final-year medical students with very low baseline correction of incorrect chest compressions. We would expect health care professionals, i.e., paramedics and emergency care physicians, to show a higher recognition rate of incorrect chest compressions at baseline. Thus, our observations are limited to resuscitation beginners, and are not necessarily applicable to more qualified staff members. Furthermore, the training setting and randomisation algorithm limit our study. This combination resulted in a slightly unequal randomisation (31 students in the CRM training vs. 26 participants in the ethics training). The strengths of our study include the prospective, blinded design, as well as the participation of team members that performed standardised incorrect chest compressions. Thus, we could evaluate every participant individually and reduce the risk of group bias.

## Conclusion

There is preliminary evidence that even a short CRM training program can result in a significant increase in corrections of overly shallow and/or fast chest compressions in a simulated scenario. As these chest compression parameters are linked to an increased patient survival, our intervention could potentially be useful to increase patient survival. Further patient-based studies are necessary to assess the impact of our intervention on real resuscitations, performed by physicians, paramedics and/or first responders.

## Additional files

Additional file 1: These are the presentations the participants received. (PDF 2116 kb)
Additional file 2: The progress for the error correction from pre- to post-measurement is displayed. The table shows the amount of scenarios in the post-measurement where participants have shown a deteriorated error correction rate, an unvarying rate or an improvement. The number of improvements is significant (*p* = 0.03*, Fisher’s exact test). (PDF 469 kb)
Additional file 3: Figure S1. Percentage of chest compressions in the reference range. The resuscitations have been analysed in short 10-s frames for the percentage of chest compressions in the reference range. The line represents the mean percentage for each group over the course of the resuscitation. (PDF 232 kb)
Additional file 4: Table S2. Effects of CRM training on resuscitation-related outcomes. Leader Behavior Description Questionnaire (LBDQ), No-flow time, Adherence to Guidelines (ADH). (PDF 28 kb)
Additional file 5: Figure S2. Amount of accumulated defibrillations as percentage (inverse Kaplan–Meier graph). The marks indicate that the first shock was delivered as early as 1 min after the scenario began. There is no major difference in the time of participants who underwent their first defibrillation. (PDF 163 kb)

## Abbreviations

ADH: Adherence to guidelines
ALS: Advanced life support
Bpm: Beats per minute
CPR: Cardiopulmonary resuscitaion
CRM: Crew resource management
ERC: European resuscitation council
LBDQ: Leader behavior description questionnaire
SD: Standard deviation
