# Supplementary material for: Improved recognition of ineffective chest compressions after a brief Crew Resource Management (CRM) training: a prospective, randomised simulation study
Source: BMC Emerg Med. 2017 Mar 3;17:7. doi: 10.1186/s12873-017-0117-6 (PMC5335734; doi:10.1186/s12873-017-0117-6)
Supplement: Additional file 1: — These are the presentations the participants received. (PDF 2116 kb) [file 12873_2017_117_MOESM1_ESM.pdf]

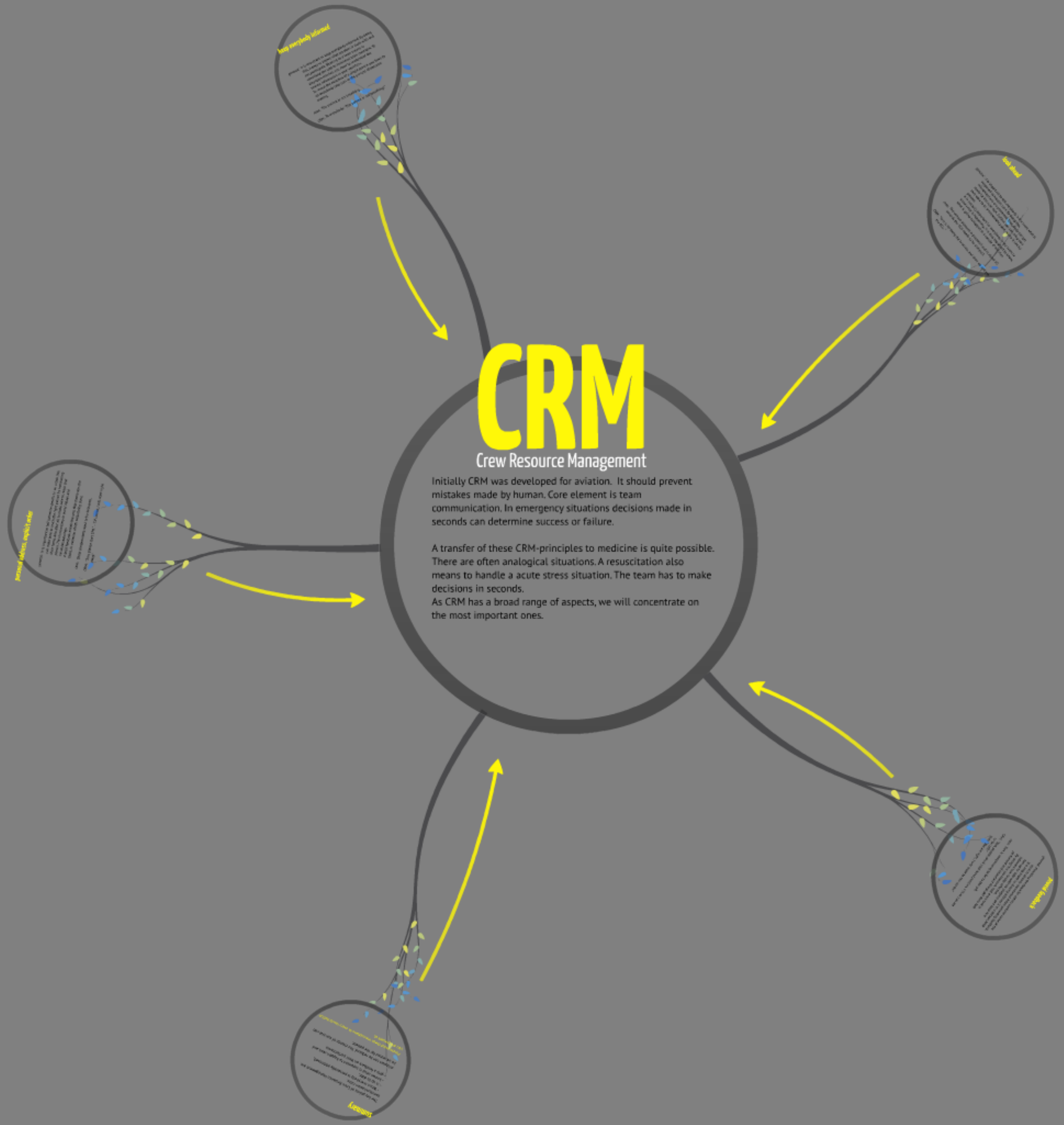
CRM
Crew Resource Management
Initially CRM was developed for aviation. It should prevent mistakes made by human. Core element is team communication. In emergency situations decisions made in seconds can determine success or failure.
A transfer of these CRM-principles to medicine is quite possible. There are often analogical situations. A resuscitation also means to handle a acute stress situation. The team has to make decisions in seconds.
As CRM has a broad range of aspects, we will concentrate on the most important ones.

CRM

Crew Resource Management

Initially CRM was developed for aviation. It should prevent mistakes made by human. Core element is team communication. In emergency situations decisions made in seconds can determine success or failure.

A transfer of these CRM-principles to medicine is quite possible. There are often analogical situations. A resuscitation also means to handle a acute stress situation. The team has to make decisions in seconds.
As CRM has a broad range of aspects, we will concentrate on the most important ones.

CRM

Crew Resource Management

Initially CRM was developed for aviation. It should prevent mistakes made by human. Core element is team communication. In emergency situations decisions made in seconds can determine success or failure.

A transfer of these CRM-principles to medicine is quite possible. There are often analogical situations. A resuscitation also means to handle a acute stress situation. The team has to make decisions in seconds.
As CRM has a broad range of aspects, we will concentrate on the most important ones.

personal address, explicit order

general: It is important to tell someone specific to do a task. You can make sure to reach the right person by mentioning their name. And after all to make sure to reach that person. This is necessary to avoid delays and misunderstandings.
A brief feedback assists the one, that distributes the tasks, to overview which tasks being done.

case: Chest compressions need to be delivered.

CRM: “Tom, please start CPR.” – “Of course, I will start right away.”

keep everybody informed

general: It is important to keep everybody informed. By doing this, everyone knows what problem is dealt with and can participate. Working as a team means to contribute the task to individual team members. To stay open-minded, you need to implement the newest information in your workflow.
To reveal the mistakes of a single person, you have to let everybody take part in the process of decision making.

case: The patient is not breathing.

CRM: To everybody: "The patient is not breathing!"

look ahead

general: It is important to tell everybody in the team what is supposed to happen next. By doing this, misconceptions are prevented. Everybody can get ready for the next task. Further you can bring your own work to an intermediate point, where it can be paused.
It is not only important for everybody in the team to know what is happening, it is also important to know, what is going to happen in a couple of minutes.

case: The venous accesses is prepared and in about 30 seconds the ECG needs to be analysed.

CRM: “Tom is finishing the iv access and then we analyse the ECG.“

provid feedback

general: Providing feedback for others contains some of the aspects already mentioned. When providing feedback, it is important to directly address it to a person, keep him up to date with his progress and keep him informed how to stay effective.
By doing this you reassure, that every task is performed and important things get done fast.

case: Tom is compressing to far to the left.

CRM: “Tom, please check your hand position. I think you are to far left.”
Tom: “You are right, I will move to the centre.”

summary

The key points of Crew Resource Management are communication rules.

- When everybody is personally addressed,
- is up to date,
- knows what is supposed to happen next and
- gets a feedback on their performance

mistakes can be reduced. The chance of survival can be increased for the patient.

Please mind these instructions in every resuscitation you are present at.

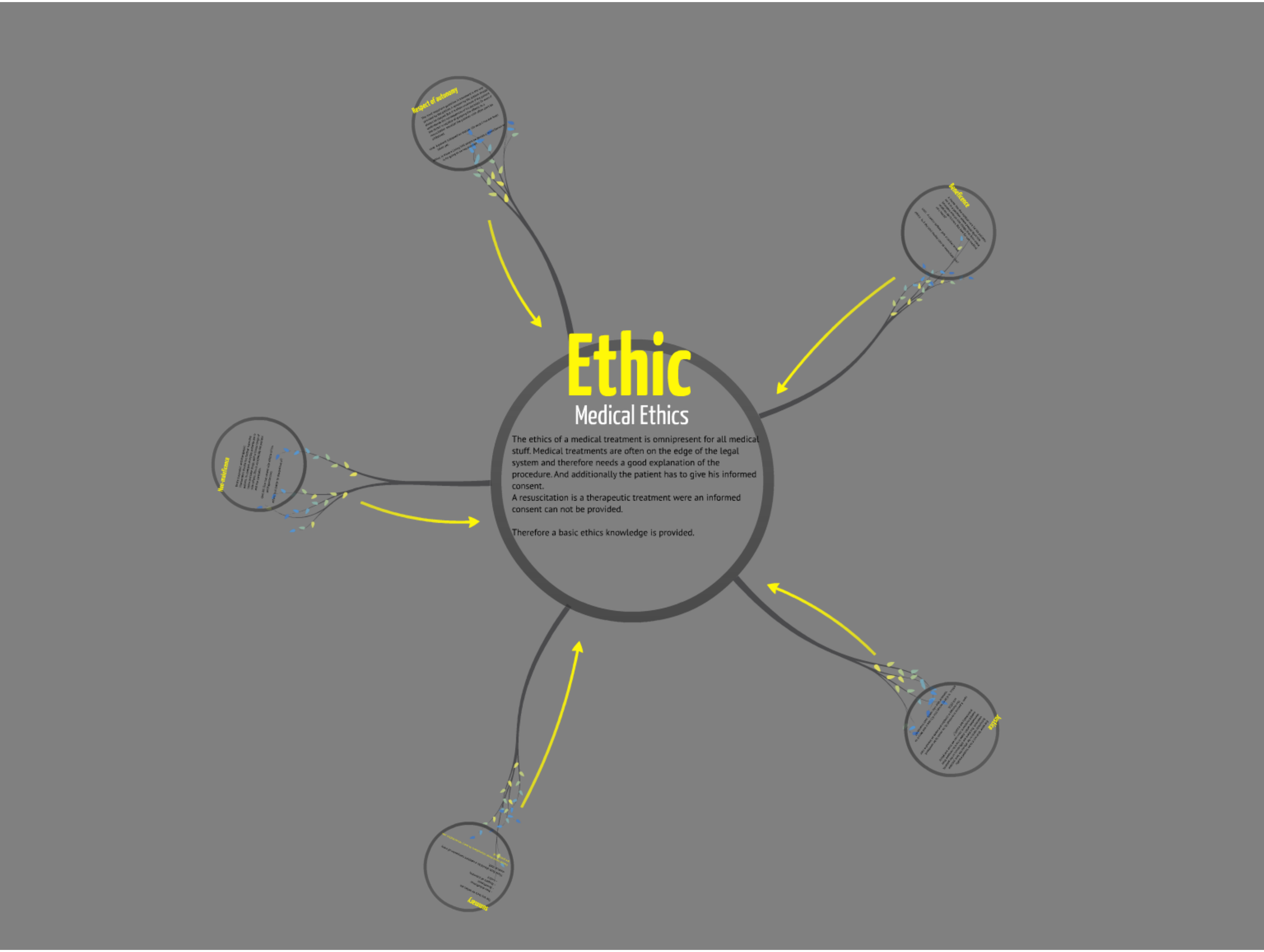
Ethic
Medical Ethics
The ethics of a medical treatment is omnipresent for all medical stuff. Medical treatments are often on the edge of the legal system and therefore needs a good explanation of the procedure. And additionally the patient has to give his informed consent.
A resuscitation is a therapeutic treatment were an informed consent can not be provided.
Therefore a basic ethics knowledge is provided.
Respect of autonomy
Beneficence
Non-maleficence
Justice

Ethic

Medical Ethics

The ethics of a medical treatment is omnipresent for all medical stuff. Medical treatments are often on the edge of the legal system and therefore needs a good explanation of the procedure. And additionally the patient has to give his informed consent.

A resuscitation is a therapeutic treatment were an informed consent can not be provided.

Therefore a basic ethics knowledge is provided.

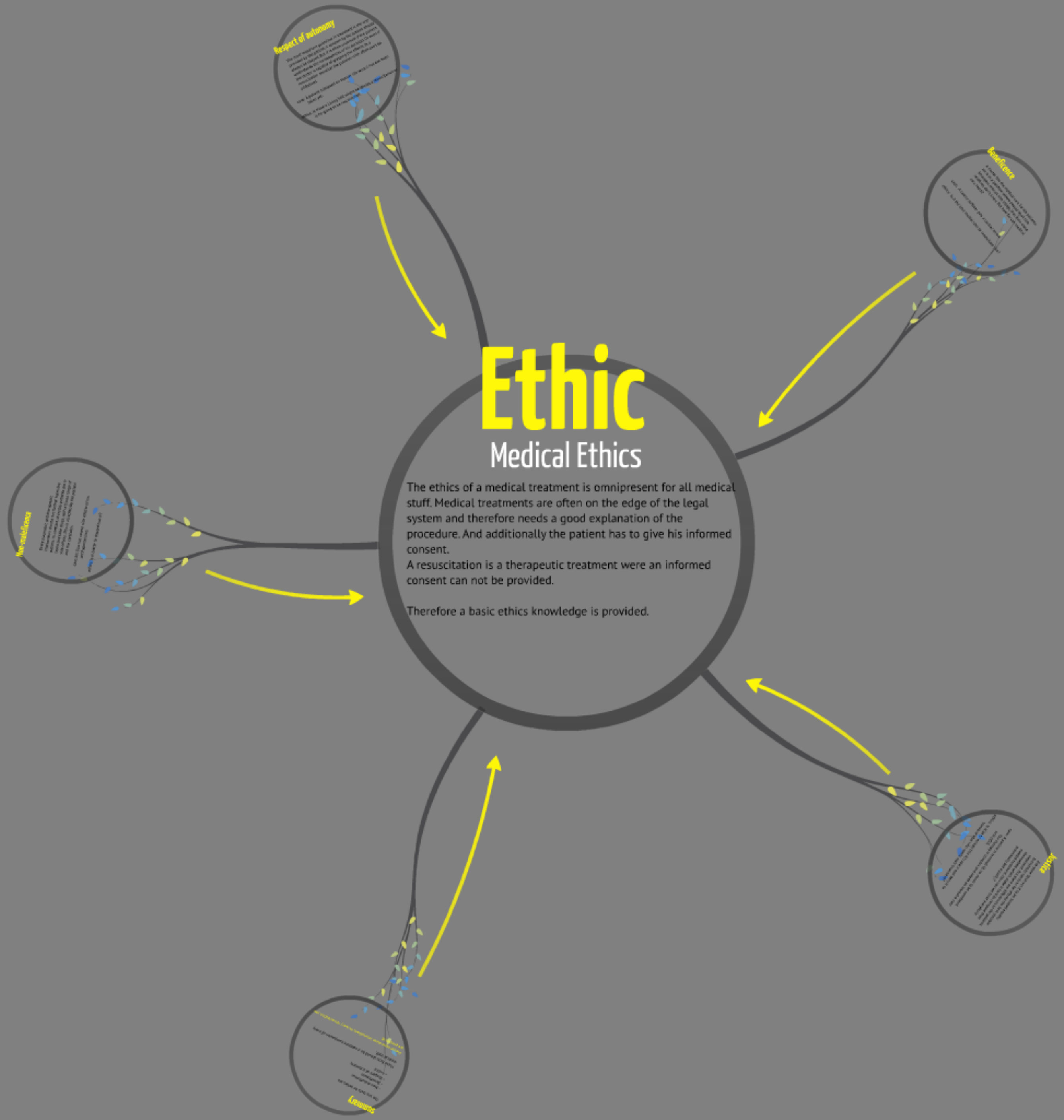

Ethic
Medical Ethics
The ethics of a medical treatment is omnipresent for all medical stuff. Medical treatments are often on the edge of the legal system and therefore needs a good explanation of the procedure. And additionally the patient has to give his informed consent.
A resuscitation is a therapeutic treatment were an informed consent can not be provided.
Therefore a basic ethics knowledge is provided.
Respect of autonomy
Beneficence

Non-maleficence

Every diagnostic and therapeutic intervention should not further harm the patient. In contrast, everyday patients are X-rayed and take drugs, with a broad range of side effects. This is accepted by the patient and the caretaker.

case: Mr. Doe has severe side effects of his antihypertensive.

ethics: Is it better to discontinue it?

Respect of autonomy

The most important guideline in treatment is the one provided by the patient. A concern by the patient should always be obeyed. But it is often uncertain if the patient understands the consequences of his decision. Or even if the doctor is capable of grasping the effects. In a resuscitation situation the patients wish often can’t be addressed.

case: A patient collapsed on station. His record has not been taken yet.

ethics: Is there a Living Will where he denies a resuscitation or is he going to be resuscitated.

Beneficence

A doctor has the medical care for his patients. He is in a position where people trust him. Strangers entrust him things, that even close relatives don't know. But how far can medical care reach?

case: A cancer sufferer gets a cardiac arrest.

ethics: Is it the best medial care to resuscitate him?

Justice

Everyone deserves it to be treated equally. Everybody needs to be offered the best possible treatment. But there are differences in the patients themselves which make it hard to compare their needed treatment. How can we treat everybody individually but equally?

case: A patient is terminal ill, he needs to be ventilated. The therapy is complex and needs an intensive care unit (ICU).

ethics: Is it ok to occupy this ICU space and deny it to someone else who needs such treatment?

summary

The key facts on ethics are

- Non-maleficence
- Beneficence
- Respect of autonomy
- Justice

These facts should be a constant companion of every medical stuff.

Please mind these instructions in every resuscitation you are present at.
