# Supplementary material for: Improved recognition of ineffective chest compressions after a brief Crew Resource Management (CRM) training: a prospective, randomised simulation study
Source: BMC Emerg Med. 2017 Mar 3;17:7. doi: 10.1186/s12873-017-0117-6 (PMC5335734; doi:10.1186/s12873-017-0117-6)
Supplement: Additional file 2: — The progress for the error correction from pre- to post-measurement is displayed. The table shows the amount of scenarios in the post-measurement where participants have shown a deteriorated error correction rate, an unvarying rate or an improvement. The number of improvements is significant (p = 0.03*, Fisher’s exact test). (PDF 469 kb) [file 12873_2017_117_MOESM2_ESM.pdf]

	Scenarios with deterioration	Unvarying scenarios	Scenarios with improvement
Ethics	1	24	1
CRM	0	23	8
