# Supplementary figures and images for: Improved recognition of ineffective chest compressions after a brief Crew Resource Management (CRM) training: a prospective, randomised simulation study
Source: BMC Emerg Med. 2017 Mar 3;17:7. doi: 10.1186/s12873-017-0117-6 (PMC5335734; doi:10.1186/s12873-017-0117-6)

## Percentage of chest compressions in the reference range

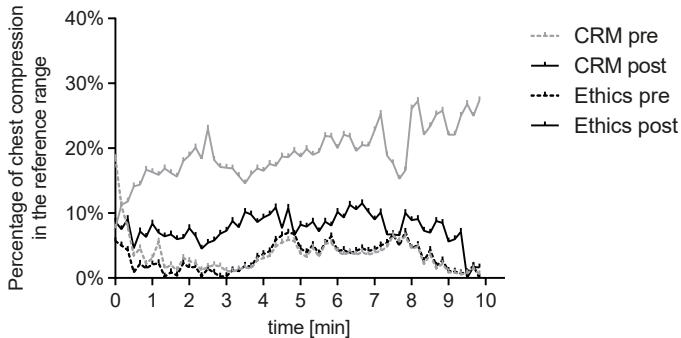

Supplement: Additional file 3: Figure S1. — Percentage of chest compressions in the reference range. The resuscitations have been analysed in short 10-s frames for the percentage of chest compressions in the reference range. The line represents the mean percentage for each group over the course of the resuscitation. (PDF 232 kb) [file 12873_2017_117_MOESM3_ESM.pdf]

## Time to defibrillation

### Inverse Kaplan-Meier Graph

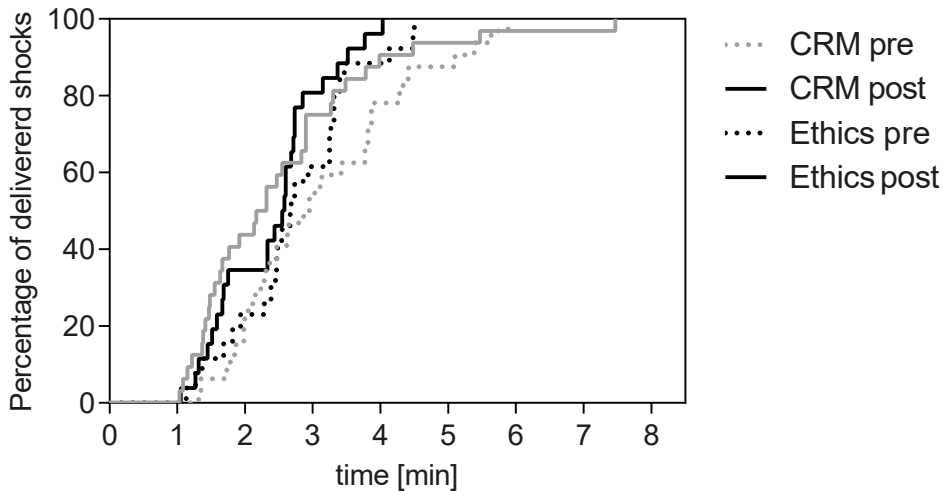

Supplement: Additional file 5: Figure S2. — Amount of accumulated defibrillations as percentage (inverse Kaplan–Meier graph). The marks indicate that the first shock was delivered as early as 1 min after the scenario began. There is no major difference in the time of participants who underwent their first defibrillation. (PDF 163 kb) [file 12873_2017_117_MOESM5_ESM.pdf]
