# Supplementary material for: Improved recognition of ineffective chest compressions after a brief Crew Resource Management (CRM) training: a prospective, randomised simulation study
Source: BMC Emerg Med. 2017 Mar 3;17:7. doi: 10.1186/s12873-017-0117-6 (PMC5335734; doi:10.1186/s12873-017-0117-6)
Supplement: Additional file 4: Table S2. — Effects of CRM training on resuscitation-related outcomes. Leader Behavior Description Questionnaire (LBDQ), No-flow time, Adherence to Guidelines (ADH). (PDF 28 kb) [file 12873_2017_117_MOESM4_ESM.pdf]

Author, Year	CRM content	Study design	Outcomes	Effects in the CRM training group
**Cooper, 2001[25]**	Team coordination, communication, leadership skills. Theory, video and discussion. 75 minute	– prospective<br>– randomized<br>– controlled	LBDQ	LBDQ score significantly higher
**Fernandez-Castelao et al. 2011[13]**	Team coordination, communication, leadership skills. Theory, video and discussion. 90 minutes	– randomized<br>– controlled	No-flow time	No-flow time significantly shorter
**Hunziker et al. 2010**	Four practical leadership instructions<br>1. Decide what to do<br>2. Tell your colleagues what they should do<br>3. Make short and clear statements<br>4. Ensure adherence to algorithm	– prospective<br>– randomized<br>– controlled	Hands-on time	Hands-on time significantly longer
**Fernandez Castelao et al. 2015[26]**	Team coordination, communication, leadership skills. Theory, video and discussion. Modified for the team leader<br>90 minutes	– prospective<br>– randomized<br>– controlled	No-flow time<br>Adherence to Guidelines (ADH)	No-flow time was shorter, but not significant<br>ADH significantly higher
**Hunziker et al. 2013[27]**	Stress coping strategies<br>10 minutes	– prospective<br>– randomized<br>– controlled	Hands-on time	Hands-on time was longer, but not significant
